# A qualitative report on experiences of participants in the young innovative leadership program

**DOI:** 10.1186/s12909-024-05033-w

**Published:** 2024-01-17

**Authors:** Atefeh Vaezi, Mohadeseh Khoshgoftar, Zahra Teimouri-Jervekani, Shaghayegh Haghjooy Javanmard

**Affiliations:** 1 Cancer Prevention Research Center, Isfahan University of Medical Sciences, Isfahan, Iran; 2 Health Education and Health Promotion, Student Research Committee and Department of Health Education and Health Promotion, School of Health, Isfahan University of Medical Sciences, Isfahan, Iran; 3 Cardiac Rehabilitation Research Center, Cardiovascular Research Institute, Isfahan University of Medical Sciences, Isfahan, Iran; 4 Department of Physiology, Applied Physiology Research Center, Cardiovascular Research Institute, Isfahan University of Medical Sciences, Isfahan, Iran

**Keywords:** Leadership, Students, health occupation, Change management, Education

## Abstract

**Background:**

Leadership in health system is a universal challenge. The Young Innovative Leadership Program (YILP) designed for undergraduate and postgraduate medical sciences students, implemented at the Isfahan University of Medical Sciences, aimed to cultivate leadership capacities through a 16-week training program. This program comprises ten modules covering innovation, change leadership, and management skills, with mentor-facilitated group discussions. This study aimed to provide a qualitative report of the experiences of participants in the YILP.

**Methods:**

A qualitative study was conducted in 2022, three months after the end of the training program, to investigate the participants’ perspectives. Data was extracted through in-depth, semi-structured interviews with 14 participants.

**Results:**

In this study 14 undergraduate and postgraduate medical sciences students who had participated in the YILP the previous year were included. Four main categories emerged from the interviews: “emergence of new horizons”, “values as beacon”, “an expanded toolbox”, and “program’s structure: a learning atmosphere”.

**Conclusions:**

The results of our study indicated that medical science students would benefit from leadership development programs. In this regard, the framework utilized to implement YILP could serve as a role model.

## Background

Healthcare providers, particularly Physicians, are expected to possess a diverse set of skills and competencies to effectively serve as community leaders, managers, communicators, caregivers, and decision-makers [1, 2]. This multifaceted role requires collaboration within interdisciplinary teams and assumes a leadership position within healthcare systems. In another word, the healthcare system relies on its health professionals to assume leadership roles within health organizations and communities, all with the overarching goal of promoting health. In this regard, the health system is responsible for preparing health professionals for this responsibility [3]. Consequently, as with any other profession, a lack of familiarity with leadership and management roles, responsibilities, and skills can create a challenging work environment, leading to undesirable outcomes for patients, hospitals, the health system, and physicians [4].

Plsek et al., argue that effective healthcare organization and delivery do not solely depend on specific targets or process control. Instead, changing an organization involves harnessing the creativity and organizing ability of staff and stakeholders through principles like generative relationships, minimum specifications, positive use of change attractors, and a constructive approach to variation, especially in moderately creative and agreement-driven areas of practice [5]. Considering the complexity of healthcare systems, there exists heterogeneity in defining leadership in health systems [6]. However, core competencies and skills for healthcare system leaders typically encompass collaboration, innovation, creativity, as well as the ability to mentor and motivate others [7].

The absence of a dedicated space in medical science education curricula poses a significant obstacle in preparing undergraduates for potential future leadership responsibilities. Literature on leadership development programs in medical education has substantially increased over the past two decades [8]. However, most studies focus on high-income countries, and few medical universities globally have integrated leadership courses into their medical sciences students’ curriculum. To the best of our knowledge, only three studies have described leadership development programs for health professionals in Iran. The first study comprised seven brief training courses for physicians and health managers [9]. The second involved a two-day workshop focusing on leadership for nurses [10]. The third consisted of a fellowship program aimed at district health managers [11]. Notably, these programs did not target undergraduate or postgraduate students.

In response, we developed the Young Innovative Leadership Program (YILP) to cultivate leadership skills and equip future health professionals for leadership roles. This study aims to present the experience of participants who attended the YILP- along with a brief overview of the program design- which was run at the Isfahan University of Medical Sciences for undergraduate and postgraduate medical sciences students.

## Methods

### Design and setting

This qualitative descriptive study was conducted between January and April 2022, approximately three months following the conclusion of the courses. Its objective was to document and present the experience of participants in a leadership training program. The study was conducted using conventional content analysis [12]. First-level codes were directly derived from interview transcripts, followed by the formation of subcategories and categories through grouping the codes. This systematic process of classification, alongside the identification of both obvious and hidden patterns and themes [13], contributed to a comprehensive understanding of the phenomenon under investigation [14].

This study received approval from the Ethics Committee of the Isfahan University of Medical Sciences (IR.MUI.MED.REC.1399.859). Prior to participation, participants were informed about the study objectives. Data were collected, analyzed, and reported anonymously. To ensure the confidentiality of the interviewees, each participant was assigned a unique code.

### The training program

The YILP was developed for undergraduate and postgraduate medical sciences students to train in innovative leadership. Further details regarding the program’s content and sessions are presented in Table 1.

The program was executed from January to September 2021 and encompassed a total of 16 weeks of training sessions, a week for mid-program wrap-up, three weeks for holidays, and an additional 16 weeks allocated for designing, implementing, and reporting final projects, with all participants engaged in the program simultaneously.

#### Content and structure

The YILP consisted of four primary components: innovation, change leadership, management skills, and innovation in the health sector. These overarching components were covered across ten modules, each lasting one to two weeks. The modules included synthesis and networking, team building, value creation and design, organizational engagement, innovative thinking, innovative reform in the health sector, leader as a mentor, change leadership, system thinking, and persuasion and influence. Each week commenced with a two-hour lecture, followed by a two-hour group discussion session led by a mentor on the third day. At the week’s conclusion, participants were required to submit a reflection paper based on their experiences. After the fourth module, a mid-program wrap-up session was conducted to facilitate connections between modules and contents. Participants were tasked with completing an end-of-course project and submitting a final reflection paper for grading. The projects were evaluated by the education committee using a Likert scoring scale based on the application of learned concepts into the projects. Students attending at least 14 out of 16 sessions and scoring a minimum of 80/100 were certified as having successfully completed the program.

#### Team structure

The YILP team comprised an education committee, program manager, and mentors, each with specific responsibilities. The education committee, consisting of the vice-chancellor of research, program organizer, program manager, and program designer, oversaw participant selection, program implementation, monitoring, and evaluation of the final projects. Mentors, selected from experienced faculty members who had been working closely with students, underwent a 3-hour mentoring/facilitation workshop to understand their roles, responsibilities, and program framework. They facilitated group exercises during discussion sessions as instructed by the lecturer. The program manager, an expert in leadership training programs, supported lecturers, mentors, and students, evaluated weekly reflection papers, provided feedback, and guided students through their final projects.

#### Application and participation

Students applied by submitting a motivation letter including inquiries about their leadership experiences, examples of leading teams, community responsibilities, and plans for initiating change. They also need to submit two recommendation letters from senior supervisors explaining students’ attitude and eligibility toward leadership based on their real life experiences. The education committee evaluated the applications using a Likert scoring scale, ultimately selecting 38 students out of 54 applicants from various disciplines and medical universities. Students were divided into six groups based on their leadership experience and preferences, with each group assigned a mentor.

#### Final outcome

Twenty-three students successfully completed the program, while four discontinued due to time constraints, eight declined to design the final project, and three did not meet completion criteria.

**Table 1:** The YILP’s content and timing

Module	Title	Summary of content	Duration (week)
1	Synthesis and networking	The significance of innovation and improvement techniques Skills required for innovation Key concepts for creating a successful network	2
2	Team building	principles of teamwork	1
3	Value creation and design	The pyramid of values Value design that meets needs Design process	2
4	Organizational engagement	Engaging the workforce Shared value in an organization Motivation	2
5	Innovative thinking	Innovative thinking and improvement techniques	1
6	Innovative reform in the health sector	Technology and the future of healthcare Health technology trends Digital health transformation	2
7	Leader as a mentor	What is mentoring? How to be a mentor? Skills and tools required by a mentor	2
8	Change leadership	Models of change leadership in an organization Different aspects of the change process Pragmatism and improvisation	2
9	System thinking	Mental model Decision making Being a system thinker	1
10	Persuasion and influence	Interaction with others How to make a win-win connection	1

### Data collection and study participants

This study focused on participants of YILP. To ensure comprehensive and valuable information, the study population was selected for interview using purposive sampling method. As the program consisted of six separate groups, one participant was chosen initially from each group and then they were asked to introduce another participant who might provide diverse perspectives. Data saturation [15], as indicated by no new information emerging, was attained after twelve interviews. To confirm data saturation, two additional interviews were conducted, which did not yield new codes or categories. Consequently, 14 interviews were analyzed for the study.

The data collection method involved semi-structured interviews. This type of interview was chosen due to its adaptability and potential to explore responses in depth. Interviews commenced with broad, open-ended questions such as “describe your experience attending this program” or “describe the gains acquired from the program”. Exploratory questions, including “please elaborate on this aspect” or “could you provide more clarity on that point?” were utilized to delve deeper into the findings. At the conclusion of each interview, interviewees were encouraged to contribute any additional insights. Interviews were conducted either in-person or online according to interviewees’ preferences, lasting between 30 and 60 min, and were recorded with participants’ consent.

### Data analysis

The textual data analysis was conducted concurrently with data collection, following the three-phase method outlined by Elo & Kungas [16]. Immediately following each interview, the audio was transcribed verbatim to gain a comprehensive understanding and immerse in data. For the coding process, semantic units such as words, sentences, or paragraphs containing essential points were identified. These semantic units were then condensed into shorter phrases representing first-level codes, considering both their apparent and implicit concepts. Subsequently, continuous comparisons of these codes led to the establishment of subcategories and larger categories based on their differences and similarities. Distinct categories emerged, and new labels were assigned as the separation of codes progressed, either fitting into existing categories or forming new ones. Throughout this iterative process, categories underwent multiple subdivisions, leading to a more extensive classification until the fundamental concepts were elucidated. Each new code was carefully compared with previous codes and categories to ensure its appropriate placement within the existing framework or the creation of a new category, ultimately resulting in the extraction of core concepts.

### Rigor

The Lincoln & Guba method was employed to ensure the validity and reliability of the data [17]. Credibility, transferability, dependability, and conformability were considered in the present study to respect the rigor of data and trustworthiness. All stages and processes of the study and reports were precisely recorded, and the best effort was made to record participants’ narrations accurately. The researchers engaged with the participants for more than a year to ensure data credibility. To increase reliability, researchers directly communicated with participants to collect data. Also, interviews transcripts and the list of subcategories and categories were checked by qualitative research experts. In addition, primary codes obtained during the interviews were confirmed by the participants through peer review to ensure coding. Two academic members, well acquainted with qualitative research, conducted external reviews.

Participants and researchers engaged in peer debriefing to ensure confirmability. Member checks were used to compare the researchers’ and participants’ opinions. Their corrections were applied, and the results were then compared with other studies. By describing the characteristics of the participants, researchers attempted to facilitate the transferability of the results in other contexts. The availability of the original data for further access was considered. In addition, a stepwise process of data generation and analysis has been reported.

Ethical considerations such as attaining informed consents, explanation of confidentiality and anonymity of the interviews, participants’ right to withdraw the interview at any time were respected.

## Results

In this study, 14 students participated, comprising seven females and seven males, with 11 successfully completed the program. Among them, five were postgraduate students, while the remaining participants were undergraduates. All study participants had prior experience in leadership across different levels. They represented diverse fields, including medicine, pharmacy, medical engineering, and public health. Further details regarding the participants’ characteristics are presented in Table 2.

**Table 2:** The characteristics of the study participants

code	Age	Gender	Education level
P1	22	Female	undergraduate
P2	21	Male	undergraduate
P3	20	Female	undergraduate
P4	25	Female	postgraduate
P5	21	Male	undergraduate
P6	24	Male	undergraduate
P7	29	Male	postgraduate
P8	24	Female	undergraduate
P9	28	Male	postgraduate
P10	23	Female	postgraduate
P11	19	Male	undergraduate
P12	25	Female	postgraduate
P13	25	Male	undergraduate
P14	24	Female	undergraduate

In this study four categories including “emergence of new horizons”, “values as beacon”, “an expanded toolbox”, and “program’s structure: a learning atmosphere”, and 13 subcategories emerged. Details are presented in Table 3.

**Table 3:** Coding of interviews

Initial codes	Subcategories	Categories
Going beyond stereotypes	Breaking boundaries	Emergence of new horizons
Going beyond your safe margin		
Expansion of the horizons		
Expanding the horizons, a way to go deep		
New neural networks		
Start the journey	Visioning the future	
More ways to go		
More things to learn		
beyond the mental boundaries		
I am supposed to make a change		
Going beyond the self	Self and beyond	Values as beacon
In peace with self		
New understanding of myself		
Peoples are important	Peoples are capitals	
Looking through others’ glasses		
Others are as important as me		
Growing together, no competition	The power of working together	
Strengths of us		
Innovation is a necessity for survival	The art of innovative leadership	An expanded toolbox
Leadership as a lifestyle		
The power of managing others		
Flexibility is a must	Soft skills	
To be a good listener		
Dialoging		
keep the dialog routes wide open		
Prejudice is a trap		
The art of deep watching		
Expansion of the network and communication		
Growth mindset	Growth mindset	
Problems are catalyzers of growth		
Let’s grow together		
More organized	Permanent and continuous attempts	
No more trial and error		
Perfectionism is an obstacle to pragmatism		
Pragmatism		
Not afraid of trying		
Learning by doing		
Thinking out of box	The ability of system thinking	
Thinking as a whole		
Importance of previous experience may lead to better takings	The importance of previous experience	Program’s structure: a learning atmosphere
An opportunity to use learned knowledge		
A confirm on previous knowledge		
The final project: a window to new understandings	The content and context of the program	
Modules were interconnected		
Interconnected content		
an opportunity for networking in the program		
Reflection papers a way to deepen the knowledge		
A wage vision at the beginning	Strength and weaknesses	
No explain on the final project		
Virtual vs. in-person		
I feel that I was valuable in this program		
Closed supervision		
multidisciplinary teams		
Learning loop		
These courses are a must		

### Emergence of new horizons

Our participants expressed a shared belief that their participation in the YILP opened up new horizons for them. They generally recognized the necessity to surpass their limitations and preconceptions. Within this category, we identified two subcategories. The first subcategory, “breaking boundaries” emerged as participants articulated the need to delve deeper into the vast realm of knowledge. They acknowledged the importance of broadening their perspectives, realizing that while their existing knowledge offered comfort, the program highlighted the imperative to step beyond this safe margin.

*“As an innovative leader, one has to leave his safe margin, and enter new spaces… one has to communicate- even with no purpose- with new people…” #p6*.

*“After the YILP I tried to accept new tasks outside my safe area… but I chose them anyway and I am happy with the result. I am glad that a new human has been born who is not the same as the one before…” #p14*.

*“I used to utilize a framework limited to my discipline references, and all I expected was that reference… but now I learned to think further… I had a project, and I told myself, let’s think differently… if I do that or put it somewhere else… I mean… I tried to get out of my closed mind… so one step removed from the final design which resulted in cost saving… it [attending YILP] benefited me a lot…” #p4*.

The Second subcategory identified was “visioning the future”. Despite all participants having prior experience in leadership and teamwork, their focus had primarily been confined to their respective disciplines. However, this program equipped them with the visionary perspective essential for effective leadership. Our study participants emphasized the necessity for initiating change and acknowledged the need to transcend their mental boundaries to embark on this transformative journey, rather than staying within their comfort zone.

*“I was just thinking in the field of my discipline. [After the YILP], I went beyond my own field and even got deeper in my field. I found out that I could be a more useful person… I understood in which field the world is moving forward and which sectors the future of health is more dependent on.” #p5*.

### Values as beacon

The second concept emerged from the interviews highlighted the significance of appreciating values. Participants acknowledged that in their previous endeavors, their focus had primarily been on self-interest rather than considering others. They credited the YILP for fostering an understanding and application of essential leadership values. They now perceive the interdependence of self and others, realizing that working collectively while considering others’ beliefs and concerns yields substantial power. Within this category, three subcategories emerged. First, “self and beyond”, where participants expressed a newfound understanding of themselves, recognizing the importance of acknowledging not only their own needs but also those of others.

*“We learned to be kind to ourselves… and to pay attention to our interests and not to suppress them. We understand that our talents should be appreciated even though it does not lay in our field of expertise… appreciating our talents could change us into a more effective human being…” #p8*.

Second, “people are capitals”, emphasized the significance of valuing others’ perspectives. Participants recognized the value of empathizing with others’ viewpoints and acknowledged the potential for enhanced performance in such an environment. Reflecting on past experiences, some participants attributed unsuccessful teamwork to neglecting others’ concerns.

*“When communicating with others, one has to understand others’ qualitative world… in this case, it would be an effective communication. I used to think about my own qualitative world all the time… my own needs and priorities… that’s why my communications were not useful in the workplace!” #p12*.

Third, “the power of working together”, marked the culmination of the previous subcategories. Our participants acknowledged that strength is derived from collaboration with others and emphasized the importance of accepting others as much as they accept themselves. They advocated for prioritizing collective growth over competition, believing that focusing on mutual advancement could be more beneficial.

*“When we are working as a team, it is much better to grow together… I mean there is no need to grow in the same way… a teammate could think differently, but it is important to grow together…” #p10*.

### An expanded toolbox

One of the primary goals of the YILP is to cultivate leaders’ soft skills and mindsets, which our participants perceived as gaining new knowledge and an expanded toolkit. This category encompasses five subcategories. The first subcategory “the art of innovative leadership”, emerged as participants stressed the importance of understanding innovation for an improved life and emphasized leaders’ need to employ innovative instincts in interpersonal interactions and managing unforeseen circumstances.

*“Leadership, to me, is not just a series of principles used to achieve great goals with a group of people, but is an art for a better life… an art that could be cultivated with practice and repetition… in my idea, leadership is a lifestyle” #p1*.

*“Today, considering the artificial intelligence and the events that take place in health; we need effective and sound innovations… hence, we need such programs which enhance innovation and creativity… so that we can use them at the right time…” #p2*.

The second subcategory, “soft skills”, encompassed the participants’ cataloged acquired skills, including flexibility, attentiveness, communication, keen observation, and the ability to broaden communication networks.

*“This program gave us different gadgets, and now we have various practical tools in our backpack. Of course, we may not have learned all the skills needed to use each of them accurately and completely, or our tools may be blunt, but we know when to use each of them, and I am sure that we will choose, sharpen, and use the appropriate ones just in case…” #p8*.

*“Starting a new discussion, learning by doing, bringing stakeholders together, empathy, storytelling, leverage our network. I had written down these points and stuck them to the wall of my room… I stared at the wall many times. These are important keywords which bring important meaning to my mind” #P11*.

The third subcategory, “growth mindset”, was identified essential, particularly for leaders, attributing its necessity to leadership. Participants highlighted personal responsibility for their own, their team’s, and their community’s development. They acknowledged a shift from avoiding problems to viewing them as catalysts for growth, emphasizing acceptance and resolution.

*“I used to think that all the team members should accept the leader, it has to be a peaceful environment, and there should be no conflict in the team… I used to believe that if a team has such problems, then the leader is not a good one. But YILP made me understand that good leaders let their team express themselves… yes, my attitude changed… also did my performance… I am no longer a strict leader.” #p3*.

*“In this program, I learned that all team members should put time for themselves and for upgrading their own skills so that to be able to carry out a project. Everyone should benefit from an opportunity to grow… and in this regard a leader could help create this opportunity for his team.” #p13*.

“Permanent and continuous attempts”, the fourth subcategory, revealed enhanced work organization attributed to the program. Participants recognized prior familiarity with these concepts, but attending YILP reinforced their confidence in previously learned lessons. Instances of project hesitance due to perfectionism were shared, with participants now understanding pragmatism in embracing potential failure.

*“Indeed, I reminded myself what we have learned in change leadership… not to be afraid and start the project, and learning by doing, etc. it helped me to complete the practical exercises in the hospital quicker than my peers…” #p1*.

The fifth subcategory, “the ability of system thinking”, was perceived as a crucial program benefit by participants. They highlighted this mindset as an invaluable tool for effective team management. Learning to think beyond conventional constraints and avoid tunnel vision when leading teams in confined environments were emphasized as key takeaways from the program.

*“If we suppose that the medical trend is to be changed, one thing that is definitely necessary is the module on system thinking… I think that was essential. Because in the process of change, we must anticipate unexpected events… and to be a part of this change, we must be able to think and have critical thinking and system thinking skills; so that to be able to be present in the stream… I think it will be very necessary.” #p5*.

### Program’s structure: a learning atmosphere

One objective of the interviews was to assess the strengths and weaknesses of the program, resulting in the classification of participants responses into this category and three subcategories. The first subcategory, “the importance of previous experience”, emerged from differing participants’ viewpoints on the significance of prior experience. While some believed that prior experience could enhance understanding, others argued for the program’s accessibility to all undergraduates, regardless of their leadership background. They emphasized the necessity for everyone to acquire leadership skills.

*“The individual with previous experience would know the necessity of these courses. I mean when I was in the work environment, I think it would be perfect if a manager knew these training… not only a manager, even the workforce who work in an organization at the lowest level.” #p9*.

*“The effect of this training program is different for those without any experience and those with at least 2–3 years of experience. The experienced participants would benefit more from lecturers, and they would have a more practical understanding of the courses.” #p7*.

The second subcategory was “the content and context of the program”. It involved discussion on program modules and the contents. Participants highlighted how the program’s context facilitated the expansion and application of their knowledge. They found value in the reflection papers, which encouraged consideration of real-world scenarios and application of newfound knowledge. Participants initially struggled to connect module contents but later integrated them while completing the final projects.

*“I did not notice the order and coordination between modules at the time of the program, but after completion of the program it become understandable to me… at the end of the program, when we had to present our project, I overcame all my fears and challenges; I managed a project and implemented it with the help of my team. First, you believe you have earned some resources and are using them to run your project… However, when I began my project, I realized that what I had previously learned served as a foundation for learning other things” #p10*.

*“[The YILP] provides an environment to practice all that we have learned… administering, networking, persuasion, and influence techniques to inspire others, including our teammates and authorities, to conduct our project… the final project was a good experience to administer the techniques we have learned, although I believe that we need more support in conducting the projects…” #p12*.

“Strengths and weaknesses”, the third subcategory, encompassed participants’ perceptions regarding obstacles and strengths: (1) Initially some participants had a vague vision and struggled to connect program courses. Opinions varied on the ideal approach, with suggestions for comprehensive explanations at the program’s onset or gradual module integration. (2) Virtual program delivery due to the situation with Covid-19 received mixed reviews; while some appreciated the accessibility it offered, others believed face-to-face sessions would enhance learning. (3) The closed supervision gave the participants a sense of worth, and they learned the values and the significance of others practically. (4) Several participants emphasized that they believed this program would only consist of some lectures with no opportunity for practice; however, they highlighted the program’s value in practical learning, transition from lectures to group discussions and connecting their learnings in real-life scenarios through reflection paper. (5) Additionally, some participants emphasized the importance of multidisciplinary teams. They acknowledged the broadened perspective and practical experience gained from diverse disciplines in developing final projects.

*“In lecture sessions, different lecturers with expertise in the field contribute to the scientific content of the program. Also, homework and teamwork we had in first sessions were very impressive, and we learned a lot doing them, but they became less important through the final sessions and lost their influence and strengths…” #p5*.

*“Fieldwork and teamwork on Mondays maintain the dynamism and vitality of the program and help us overcome the limitations of being virtual. Relevant and creative exercises in Monday sessions were the most important strengths of the YILP. Furthermore, the formation of smaller groups with a supervisor was very effective. Overall, the program was good but had a big problem; the projects were defined at the end of the program; they had to be defined at the beginning. In that case, we would be able to update our project through the sessions. Maybe it would be better to ask for a proposal from the participants in the first place.” #p7*.

## Discussion

This study delves into the perspective of students who participated in the YILP, a leadership development program designed and executed at the Isfahan University of Medical Sciences for both undergraduate and postgraduate medical sciences students. The findings of this study delineate four main categories, including “emergence of new horizons”, “values as beacon”, “an expanded toolbox”, and “program’s structure: a learning atmosphere”.

Overtime, the focus of leadership training programs has evolved from preparing select physicians for organizational leadership roles to encompassing all physicians as everyday life leaders. This shift necessitates the incorporation of leadership training programs into the medical curriculum [18, 19]. Nevertheless, challenges persist regarding the design and delivery of leadership courses for medical sciences students. Bekas reviewed relevant literature and concluded that foundational elements of leadership training programs encompass experiential learning, reflective practice, action learning, and mentoring [20]. Competency-based programs are hailed as effective tools for imparting fundamental leadership concepts [21]. Competencies include general and role-specific knowledge and skills that a leader requires, including critical thinking, decision-making, problem-solving, inspiring others, and teamwork among other essential skills [22]. YILP was purposefully designed to facilitate the practical application of acquired knowledge. Participants’ experience aligns with the findings of the Diaz-Monsalve study, advocating for the integration of practical elements alongside theoretical courses in leadership training [23]. YILP employs a participatory training method, offering a favorable environment for participants to apply their learning to real-life situations.

Walsh et al. advocate for the introduction of leadership development programs across various disciplines, commencing at the undergraduate level and persisting throughout one’s career [24]. A recent leadership training program targeting individuals aged 30 and older, demonstrates the significance of early career leadership training [25]. Some believe that leaders are inherently born as such and do not require any formal education or training. However, it has been shown that they will face an unanticipated crisis after a brief period of success. Evidence suggests the importance of acquiring requisite skills and knowledge prior to assuming leadership roles [26]. Reliance solely on traditional qualifications for leaders, such as age or academic performance together with lack of formal education may lead to emulation of predecessors’ practices, negatively impacting communities [27]. There is a positive correlation between leadership development programs and enhanced leadership outcomes. Geerts et al. demonstrated that these programs should encompass contents regarding decision-making, communication, teamwork, and career growth skills [28]. In this regard, discussions arose regarding the advantages of prior leadership experiences. All YILP participants possessed varying degrees of leadership exposure, albeit none had attained organizational-level leadership roles. Within our study population, divergent opinions surfaced among participants. While some emphasized the significance of previous experience in enhancing comprehension, others advocated for universal access to the program, irrespective of leadership background.

The primary objective of YILP was to transcend the notion of leadership as a mere set of prescribed rules. The program aimed to interlink diverse concepts through lecture courses, reinforce these competencies via mentor-assisted sessions and problem-based learning activities, encourage reflection on past experiences, and enable the application of acquired knowledge in real-world scenarios through end-of-course projects. When designing a program, it’s important to include the topic, the rationale behind the topic and the skills participants will acquire, details about the speakers and their expertise, and ways in which learning can be applied post-event [29]. The participants in our study acknowledged that YILP imparted them with valuable leadership skills and perspectives. Yet, they initially found it challenging to grasp the interconnectedness of various skills taught in the program. They highlighted that engagement in practical field tasks and guidance from mentors significantly aided their learning process. However, despite the final project offering a comprehensive overview, eight participants opted not to adhere to this aspect, finding it difficult to follow through.

YILP delivered virtually due to the constraints imposed by the COVID-19 pandemics. Executing YILP as a virtual course facilitated participation of students from other universities, however some of our participants believed that they might benefit more if the course was executed in-person. The effectiveness of online and in-person workshop seems to be similar [30], however, best results could be obtained from in-person workshops, when engagement, communication and networking, and creativity are important [31].

There were two structural elements in YILP among others, which helped participants to relate real-life experiences: the multidisciplinary base of the participants and mentoring. Interdisciplinary education, as witnessed in the YILP’s diverse participant base, extends further than just developing leadership skills among medical sciences students. It plays a pivotal role in nurturing a culture of collaboration, adaptability, and interdisciplinary teamwork [32]. The collaborative nature of the program encourages students to appreciate the importance of diverse viewpoints and approaches. Participants of our study recognized the imperative for leaders to collaborate with their teams, acknowledge their values and priorities, and contribute to their growth. leadership styles lean less toward authoritarianism and more toward collaboration, emphasizing support for teams, envisioning shared goals, and fostering empowerment and development [33].

Another element in the YILP was mentor-facilitated activities. Mentorship emerges as a critical aspect of leadership. Leaders should serve as mentors for their team, hence, mentor-assisted training programs providing distinct advantages [34, 35]. Participants in our study expressed gaining familiarity with these concepts through the program’s sessions and delivery method. They conveyed a sense of being guided along their learning journey. Moreover, they acknowledged that they had acquired additional skills, which, although not fully mastered, were now part of their toolkit for potential use.

Our study has encountered certain limitations. It’s crucial to note that this evaluation is based on a single execution of the course, relying solely on the participants’ perspectives. Additionally, the evaluation took place nearly three months after the program concluded, potentially skewing the results towards a more positive perspective from participants. Hence, caution should be taken in interpreting the results. We firmly believe a definitive conclusion regarding the effectiveness of this program should evolve over time. Furthermore, our study did not incorporate the viewpoint of other stakeholders, including lecturers, mentors, and the education committee. The inclusion of these perspectives could have contributed to the overall comprehensiveness of our evaluation. Lastly, due to the virtual presentation of the YILP, the results of our study preclude a definite opinion regarding the integration of this program into the educational curriculum.

## Conclusions

In this study we aimed to evaluate a leadership training program through the participants’ perspective. The participants of our study described the YILP as a valuable training program which provides them with new horizons, attitude regarding values in leadership, and an enriched toolbox in a learning atmosphere. Although the overall structure and framework of this program could be utilized as a model for the expansion of future leadership training programs, cautions need to be taken when executing the program in-person and for students without previous leadership experiences. Additionally, we believe that more comprehensive studies on the integration of leadership courses in the curriculum of medical sciences students are required.

## Data Availability

The data analyzed during the current study are available via the corresponding author.
